# Transcriptome Assembly and Profiling of *Candida auris* Reveals Novel Insights into Biofilm-Mediated Resistance

**DOI:** 10.1128/mSphere.00334-18

**Published:** 2018-07-11

**Authors:** Ryan Kean, Christopher Delaney, Leighann Sherry, Andrew Borman, Elizabeth M. Johnson, Malcolm D. Richardson, Riina Rautemaa-Richardson, Craig Williams, Gordon Ramage

**Affiliations:** a Oral Sciences Research Group, School of Medicine, Dentistry and Nursing, College of Medical, Veterinary and Life Sciences, University of Glasgow, Glasgow, United Kingdom; b Institute of Healthcare, Policy and Practise, University of the West of Scotland, Paisley, United Kingdom; c National Mycology Reference Laboratory, Public Health England South-West, Bristol, United Kingdom; d Mycology Reference Centre Manchester, University Hospital of South Manchester & University of Manchester, Manchester Academic Health Sciences Centre, Faculty of Biology, Medicine and Health, Division of Infection, Immunity and Respiratory Medicine, Manchester, United Kingdom; e ESCMID Study Group for Biofilms (ESGB)‡; Carnegie Mellon University

**Keywords:** *Candida*, antifungal resistance, biofilms, efflux pumps, gene expression

## Abstract

Candida auris has emerged as a significant global nosocomial pathogen. This is primarily due to its antifungal resistance profile but also its capacity to form adherent biofilm communities on a range of clinically important substrates. While we have a comprehensive understanding of how other *Candida* species resist and respond to antifungal challenge within the sessile phenotype, our current understanding of C. auris biofilm-mediated resistance is lacking. In this study, we are the first to perform transcriptomic analysis of temporally developing C. auris biofilms, which were shown to exhibit phase- and antifungal class-dependent resistance profiles. A *de novo* transcriptome assembly was performed, where sequenced sample reads were assembled into an ~11.5-Mb transcriptome consisting of 5,848 genes. Differential expression (DE) analysis demonstrated that 791 and 464 genes were upregulated in biofilm formation and planktonic cells, respectively, with a minimum 2-fold change. Adhesin-related glycosylphosphatidylinositol (GPI)-anchored cell wall genes were upregulated at all time points of biofilm formation. As the biofilm developed into intermediate and mature stages, a number of genes encoding efflux pumps were upregulated, including ATP-binding cassette (ABC) and major facilitator superfamily (MFS) transporters. When we assessed efflux pump activity biochemically, biofilm efflux was greater than that of planktonic cells at 12 and 24 h. When these were inhibited, fluconazole sensitivity was enhanced 4- to 16-fold. This study demonstrates the importance of efflux-mediated resistance within complex C. auris communities and may explain the resistance of C. auris to a range of antimicrobial agents within the hospital environment.

**IMPORTANCE** Fungal infections represent an important cause of human morbidity and mortality, particularly if the fungi adhere to and grow on both biological and inanimate surfaces as communities of cells (biofilms). Recently, a previously unrecognized yeast, Candida auris, has emerged globally that has led to widespread concern due to the difficulty in treating it with existing antifungal agents. Alarmingly, it is also able to grow as a biofilm that is highly resistant to antifungal agents, yet we are unclear about how it does this. Here, we used a molecular approach to investigate the genes that are important in causing the cells to be resistant within the biofilm. The work provides significant insights into the importance of efflux pumps, which actively pump out toxic antifungal drugs and therefore enhance fungal survival within a variety of harsh environments.

## Abstract (precis)

Fungal infections represent an important cause of human morbidity and mortality, particularly if the fungi adhere to and grow on both biological and inanimate surfaces as communities of cells (biofilms). Recently, a previously unrecognized yeast, Candida auris, has emerged globally that has led to widespread concern due to the difficulty in treating it with existing antifungal agents. Alarmingly, it is also able to grow as a biofilm that is highly resistant to antifungal agents, yet we are unclear about how it does this. Here, we used a molecular approach to investigate the genes that are important in causing the cells to be resistant within the biofilm. The work provides significant insights into the importance of efflux pumps, which actively pump out toxic antifungal drugs and therefore enhance fungal survival within a variety of harsh environments.

## INTRODUCTION

Fungal infections affect in excess of a billion people, resulting in approximately 11.5 million life-threatening infections and more than 1.5 million deaths annually (1). Candida auris is an emerging fungal pathogen that has attracted considerable attention because of its ability to cause infections that are difficult both to diagnose and to treat (2). It has been responsible for a number of nosocomial outbreaks worldwide through its ability to persistently colonize and be transmitted between patients and the environment (3–6). Despite the unprecedented global emergence of this organism, relatively little is known about the molecular basis of its pathogenicity and antifungal resistance phenotype. The resistance profile is well documented, with >90% of isolates intrinsically resistant to fluconazole. Resistance to other azoles, polyenes, and echinocandins has also been reported (4). Alarmingly, 41% of isolates have been shown to be multidrug resistant, with 4% demonstrating pan-drug resistance (4). Hot spot mutations in *ERG11* and *FKS1* have been identified as resistance mechanisms in azole- and echinocandin-resistant strains, respectively (7, 8).

*Candida* biofilms represent an important clinical entity associated with adaptive resistance to many antifungals and are linked to excess morbidity and mortality (9–11). Although Candida albicans is regarded as the primary biofilm-forming pathogen within the genus, there are increasing interest in and evidence for non-Candida albicans-species biofilms (12, 13), particularly those of C. auris. Clinically, C. auris has been isolated from a number of sites, including wounds, line tips, and catheters, suggestive of the organism existing within a biofilm lifestyle in the host (14, 15). We recently described the ability of C. auris to form antifungal-resistant biofilms, against all 3 main classes of antifungals (16), and yet the mechanisms underlying this phenotype remain unknown. The speed of discovery in this emerging pathogen has certainly been hindered by the lack of robust sequence information. Initial sequencing efforts provided a draft C. auris genome; however, these reads were poorly aligned to other *Candida* spp. and inconsistently annotated (17). More recently, complete and functionally annotated genome assemblies have been created, allowing the analysis of the functional capacity of the genome to be studied under clinically relevant conditions (18). Biofilm-associated resistance is a complex and multifaceted phenomenon that has been described in a number of fungal pathogens. Various resistance mechanisms exist, predominately associated with the extracellular matrix (ECM), overexpression of drug targets, and efflux pumps (19). Given the lack of understanding of biofilm formation and resistance mechanisms in C. auris, we therefore aimed to investigate these mechanisms using a transcriptomics approach.

## RESULTS

### *Candida auris* biofilms exhibit temporal antifungal resistance.

**FIG 1:**
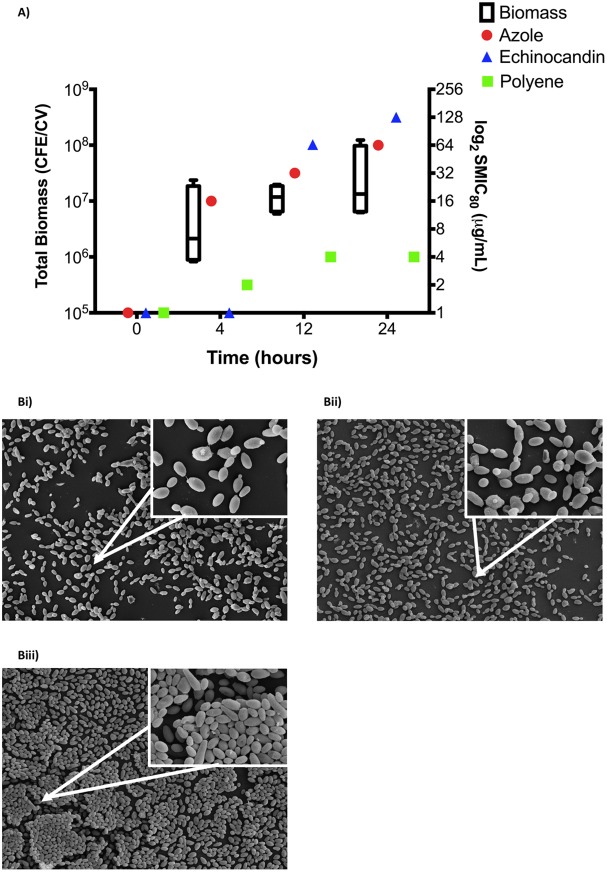
Candida auris biofilm development correlates with increased antifungal tolerance. Candida auris biofilms were standardized at 1 × 10^6^ CFU/ml and grown for 4, 12, and 24 h. Biofilm biomass was then quantified using the crystal violet assay, with the composition of biofilm cells enumerated using qPCR and represented by a box-and-whisker plot as the total biomass of four C. auris isolates (A, left *y* axis). Planktonic susceptibility testing was performed against serially diluted miconazole, micafungin, and amphotericin B concentrations using the CLSI guidelines, with biofilm susceptibility testing performed using the XTT assay and with median MIC values plotted (A, right *y* axis). In addition, biofilms were grown, fixed, and processed for SEM before imaging using a JEOL-JSM-6400 scanning electron microscope. Micrographs represent lower magnification (×1,000) and higher magnification (inset, ×5,000) of biofilms grown for 4 h (Bi), 12 h (Bii), and 24 h (Biii).

Mature Candida auris biofilms have been shown to be resistant to antifungals that are readily active against their planktonic equivalents (16). We therefore investigated the temporal effect of biofilm formation on the susceptibility to all three major classes of antifungals. As demonstrated in Fig. 1A, the maturation of C. auris biofilms was shown to correlate with decreased susceptibility to each antifungal agent. When assessed planktonically, the median MIC for the four isolates of miconazole was 1 µg/ml, that of micafungin was <0.25 µg/ml, and that of amphotericin B was 0.5 µg/ml (range, 0.125 to 0.5 µg/ml). After 4 h of biofilm development, no increases in resistance were observed against micafungin (MIC, <0.25 µg/ml); however, the median MIC increased 16-fold to 16 µg/ml (range, 16 to 32 µg/ml) for miconazole and 4-fold to 2 µg/ml for amphotericin B (range, 1 to 4 µg/ml). As the biofilm matured to 12 h of growth, 2-fold increases in median MIC were shown for miconazole (range, 16 to 64 µg/ml) and amphotericin B (range, 2 to 4 µg/ml). Interestingly, the MIC was shown to significantly increase for micafungin (range, 1 to >128 µg/ml) after 12 h. After 24 h, no further increase in MIC was observed for amphotericin B. However, both miconazole and micafungin MICs were increased 2-fold to 64 µg/ml and >128 µg/ml, respectively.

### *Candida auris* transcriptome assembly.

**FIG 2:**
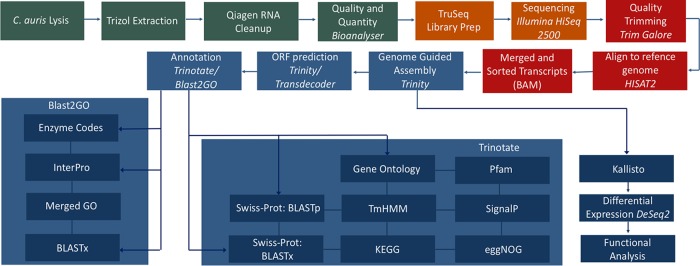
Bioinformatic pipeline for Candida auris transcriptome assembly, annotation, and analysis.

Given the temporal patterns of biofilm-associated resistance, we undertook a transcriptional profiling approach to understand the mechanisms governing antifungal biofilm resistance (Fig. 2). Sequencing of samples using Illumina HiSeq produced around 414 million single-end reads of 50-bp length. Following processing, the number of reads was reduced by 3 million through trimming and quality control stages. All sequenced sample reads were then assembled into an ~11.5-Mb transcriptome which consisted of 5,889 identified Trinity transcripts and 5,848 genes based on the longest isoform of transcripts. At least half of the assembled sequenced bases were found on contigs of a length of 3,488 bp (*N*_50_) (Table 1). The completeness and quality of the C. auris transcriptome were assessed with BUSCO (Benchmarking Universal Single-Copy Orthologs) against Ascomycota (94%), Saccharomyceta (91.4%), and Saccharomycetales (91.7%) gene sets. Very small percentages of duplicate, fragmented, and missing genes were observed in each of the gene sets (Table 2).

**TABLE 1:** Summarized statistics for transcriptome assembly of Candida auris, alignment rate of raw reads to transcriptome, and summary of Trinotate functional annotation

Category	Value
No. of reads	
Total	414,364,539
After trimming	411,626,529
Total no. of assembled bases	11,593,681
GC content, %	45.35
Total no. by Trinity	
“Genes”	5,848
“Transcripts”	5,889
Contig (bp)	
*N*_50_	3,488
Median	1,308
Avg	1,983
No. of reads aligned (%)	
1 time	393,124,946 (95.51)
>1 time	9,368,727 (2.28)
Overall	402,493,673 (97.78)
Functional annotation, no. of transcripts	
Swiss-Prot matches, BLASTx	3,200
Swiss-Prot unique proteins, BLASTx	3,176
Swiss-Prot matches, BLASTp	3,041
Swiss-Prot unique proteins, BLASTp	3,019
TMHMM	701
SignalP	202
Gene Ontology	3,085
KEGG	2,889

**TABLE 2:** Assessment of Candida auris transcriptome assembly by Benchmarking Universal Single-Copy Orthologs (BUSCO)

% genes	Ascomycota	Saccharomyceta	Saccharomycetales
Complete	94	91.4	91.7
Complete single copy	93.4	90.5	90.9
Complete duplicated	0.6	0.9	0.8
Fragmented	3.4	4.8	4.6
Missing	2.6	3.8	3.7
Total no. of genes	1,315	1,759	1,711

Identification by sequence homology searches with BLASTx function yielded annotation of 54% of Trinity transcripts and 54% of unique “genes.” Identification of protein sequences with BLASTp, against TransDecoder-identified open reading frames (ORFs) and potential coding sequences, gave functional annotation matches with 51% of the transcripts and 41% of unique “genes” (Table 1). The presence of known signal peptides, functional protein domains, and protein topology was discerned by searches against the SignalP and TMHMM databases, respectively. Of the predicted proteins, 202 sequences were predicted to have signal peptides and 701 transmembrane protein topologies were predicted.

Additional annotation was performed via the software BLAST2GO, which obtains BLAST hits that are used to retrieve and map gene ontology (GO) and KEGG terms. It also utilizes InterProScan, which acquires functional annotation of protein sequences from EBI’s InterPro databases (https://www.ebi.ac.uk/interpro/). These databases are a consortium of online databases that include PANTHER, Pfam, and SUPERFAMILY (20). Both the Trinotate and BLAST2GO annotation files are supplied as Data Set S1 in the supplemental material.

10.1128/mSphere.00334-18.5 DATA SET S1  Trinotate and BLAST2GO annotation. Download DATA SET S1, XLSX file, 12 MB. Copyright © 2018 Kean et al. 2018 Kean et al. This content is distributed under the terms of the Creative Commons Attribution 4.0 International license.

BLAST2GO searches were performed with a fungus taxonomical filter, which annotated 1,157 genes with BLAST and an additional 4,365 genes from the InterPro databases. InterPro and BLAST-derived GO terms were merged to give a total of 9,504 GO annotations assigned to 2,479 genes. These annotations were distributed among three main GO categories, biological process (3,633, 38%), cellular component (3,116, 33%), and molecular function (2,755, 29%) (Fig. S1). InterProScan was able to classify Trinity transcripts according to superfamilies based on known structures. The best-represented superfamilies were the P-loop-containing nucleoside triphosphate hydrolase (236 genes), the major facilitator superfamily (MFS) (113 genes), Armadillo-type fold (102 genes), and protein kinase-like superfamily (90 genes) (Fig. S2). From annotation against the available databases, there were 6 major enzyme classes represented, which included hydrolyases (290 genes), transferases (150 genes), oxidoreductases (88 genes), ligases (21 genes), lyases (22 genes), and isomerases (15 genes) (Fig. S3).

10.1128/mSphere.00334-18.1 FIG S1  Gene Ontology term distribution. GO-based annotation was performed by BLAST2GO and InterProScan, which functionally classified transcripts into terms associated with biological process (BP), cellular component (CC), and molecular function (MF). Download FIG S1, TIF file, 2.1 MB. Copyright © 2018 Kean et al. 2018 Kean et al. This content is distributed under the terms of the Creative Commons Attribution 4.0 International license.

10.1128/mSphere.00334-18.2 FIG S2  Superfamily distribution of assembled transcripts. Assembled transcripts were submitted to an InterProScan search against the SUPERFAMILY distribution protein database for functional classification, with 479 annotations against identified sequences. Download FIG S2, TIF file, 2.1 MB. Copyright © 2018 Kean et al. 2018 Kean et al. This content is distributed under the terms of the Creative Commons Attribution 4.0 International license.

10.1128/mSphere.00334-18.3 FIG S3  Functional characterization of potential enzyme transcripts in Candida auris. Assembled transcripts were identified by BLAST2GO via enzyme code mapping with transcripts distributed across 6 major enzyme classes. Download FIG S3, TIF file, 2.1 MB. Copyright © 2018 Kean et al. 2018 Kean et al. This content is distributed under the terms of the Creative Commons Attribution 4.0 International license.

### DE and functional annotation of C. auris biofilms.

**FIG 3:**
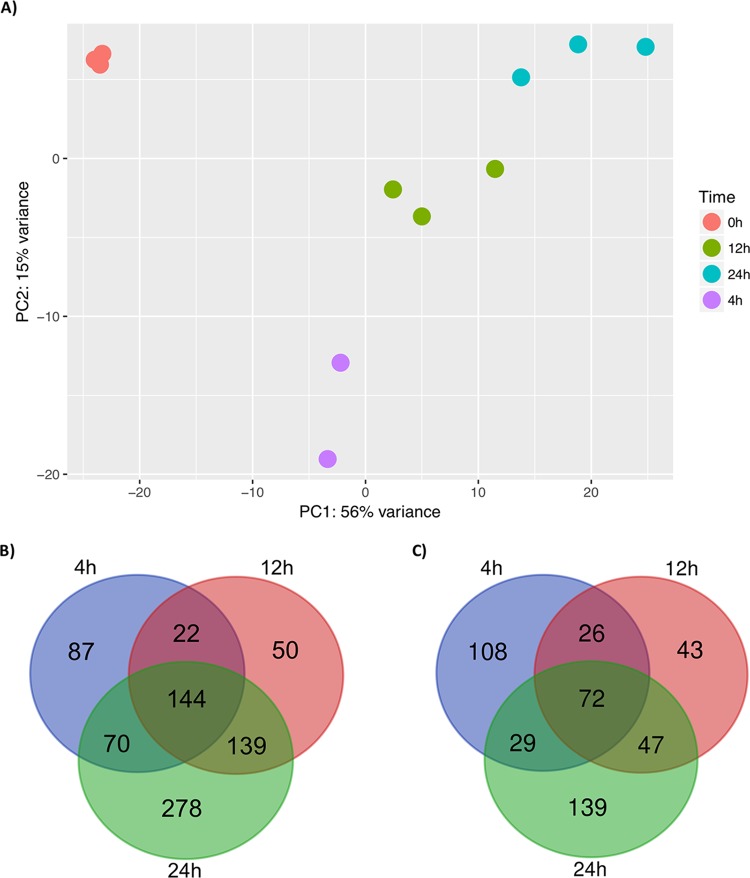
Quality control and differential expression analysis of C. auris biofilms. Principal-component analysis displays the largest variance along PC1 (56%) and the second largest variance between samples along PC2 (15%) (A). Venn diagrams of the genes upregulated (B) and downregulated (C) in biofilm time points (4, 12, and 24 h) compared to 0 h.

**FIG 4:**
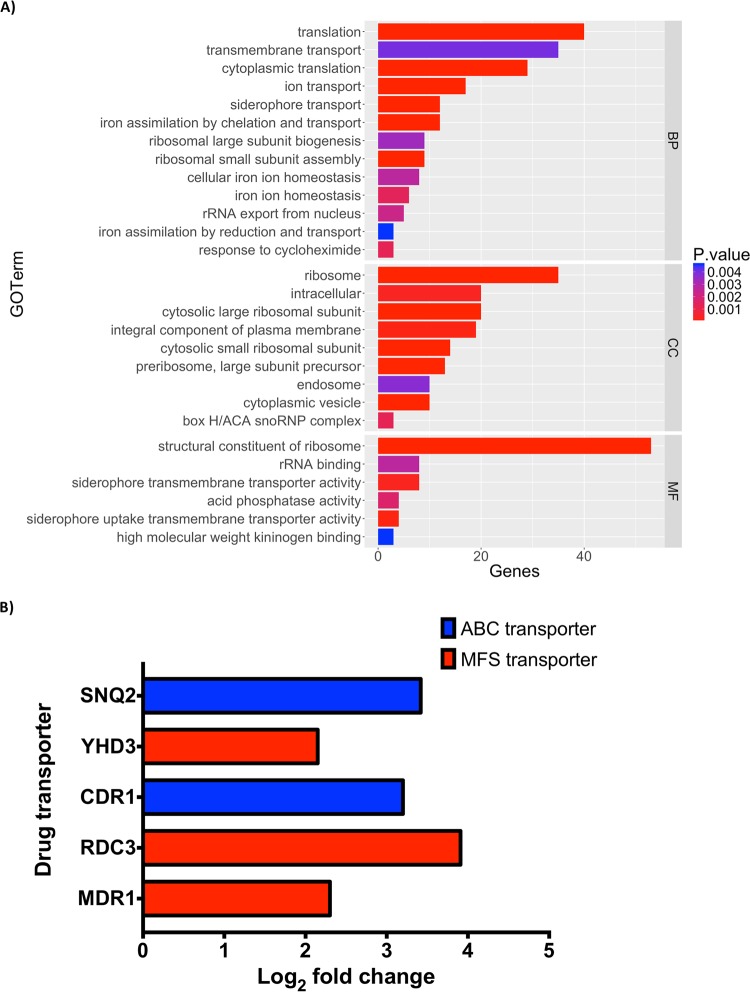
Functional annotation of differentially expressed genes reveals upregulation of drug transporters. Gene distribution of significantly upregulated C. auris genes in 24-h biofilms relative to planktonic cells, grouped into biological process (BP), cellular component (CC), and metabolic function (MF) gene ontology categories (A). Log_2_ fold change of upregulated ABC and MFS drug transporters within 24-h biofilms (B). All GO terms have a *P* value of <0.05 based upon the GOSep hypergeometric distribution test.

Differential expression (DE) analysis was performed to investigate the transcriptional changes observed with biofilm development. Multivariate analysis by principal-component analysis (PCA) demonstrates variance between the different time points; 0 h shows the greatest variance from the other biofilm time points. In addition, there is also some variance between biofilms at 4, 12, and 24 h (Fig. 3A). DE analysis demonstrated that 791 and 464 genes were upregulated in biofilm formation and planktonic cells, respectively, with a minimum 2-fold change (Fig. 3A). Phase-dependent differential expression of these upregulated genes is illustrated in the Venn diagram in Fig. 3B, with the downregulated genes shown in Fig. 3C; individual genes are described in Data Set S2. Of these biofilm-upregulated genes, selected genes involved in antifungal resistance and biofilm-associated mechanisms are listed in Table 3. Glycosylphosphatidylinositol (GPI)-anchored cell wall genes, including *IFF4*, *CSA1*, *PGA26*, and *PGA52*, were upregulated at all time points of biofilm formation, highlighting their potential role within cellular adhesion (Table 3). Two further adhesins, *HYR3* and *ALS5*, were also shown to be upregulated but only in mature biofilms (Table 3). As the biofilm developed into intermediate and mature stages, a number of genes encoding efflux pumps were upregulated, including *RDC3*, *SNQ2*, *CDR1*, and *YHD3*. In addition, *MDR1* was shown to be upregulated at the 24-h time point (Table 3). To understand the functional processes related to differentially expressed genes, a cutoff of 2-fold upregulation (adjusted *P* value of <0.05) was used for gene ontology (GO) analysis comparing planktonic cells to 24-h biofilms. The 278 differentially expressed genes were assigned to 28 GO terms with an overenrichment *P* value of <0.05, comprising 13 biological processes, 9 cellular components, and 6 molecular functions, and contained a number of differentially expressed functional categories (Fig. 4A). Included within these GO terms were transmembrane transport, within which several ATP-binding cassette (ABC) and major facilitator superfamily (MFS) transporters were highly upregulated in C. auris biofilms (Fig. 4B).

10.1128/mSphere.00334-18.6 DATA SET S2  Differentially expressed genes. Download DATA SET S2, XLSX file, 0.3 MB. Copyright © 2018 Kean et al. 2018 Kean et al. This content is distributed under the terms of the Creative Commons Attribution 4.0 International license.

**TABLE 3:** Upregulated biofilm- and resistance-associated genes

Gene identifier	Function	Fold change compared to planktonic cells (log_2_)		
		4 h	12 h	24 h
*IFF4*	Adhesion	2.29	5.01	3.62
*PGA26*	Adhesion	2.02	3.90	2.55
*PGA52*	Adhesion	2.22	2.38	2.42
*CSA1*	Adhesion	3.87	6.47	6.43
*PGA7*	Adhesion		3.94	4.82
*HYR3*	Adhesion			2.06
*ALS5*	Adhesion			3.82
*RDC3*	Efflux pump		4.29	3.91
*SNQ2*	Efflux pump		2.63	3.42
*CDR1*	Efflux pump		2.30	3.19
*YHD3*	Efflux pump		2.14	2.15
*MDR1*	Efflux pump			2.3
*KRE6*	Extracellular matrix		3.92	3.09
*EXG*	Extracellular matrix		2.69	2.26
*SAP5*	Hydrolytic enzyme			2.19
*PLB3*	Hydrolytic enzyme			2.13

### Efflux pumps play a primary role in antifungal resistance in C. auris biofilms.

**FIG 5:**
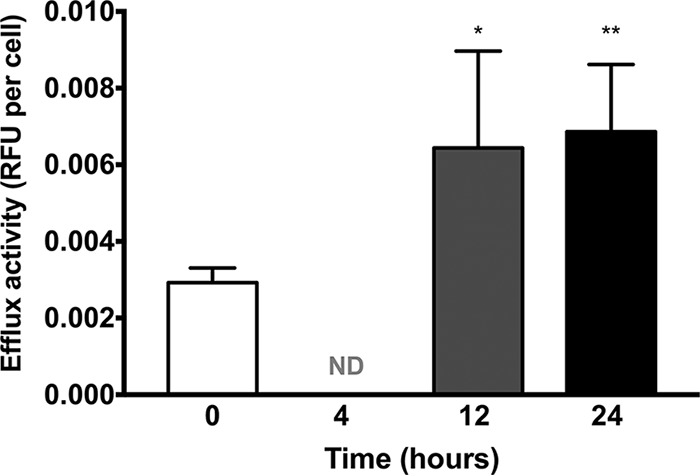
Efflux pump activity is increased in Candida auris biofilms. Candida auris biofilms were grown for 4, 12, and 24 h in black-bottomed 96-well plates. In addition, planktonic cells were standardized to 5 × 10^7^ cells/ml, all cells were incubated with 100 µg/ml of Ala-Nap, and fluorescence measurements were read at 30-s intervals over 60 min (excitation, 355 nm; emission, 460 nm). Data represent the mean + standard deviation of 4 isolates repeated on 3 independent occasions. Data presented are relative fluorescence units (RFU) normalized per individual cell. *, *P* < 0.05; **, *P* < 0.01; ND, not detectable.

Transcriptional analysis and function annotation revealed a significant upregulation of a number of drug efflux pumps, from both ABC and MFS transporters. To confirm the role of these membrane proteins within biofilms, we assessed efflux pump activity. Both 12- and 24-h biofilms exhibited increased efflux compared to planktonic cells, with 4-h biofilms below the detectable limit of the assay. Efflux from 12-h biofilms was 2.21-fold (*P* < 0.05) greater than that from planktonic cells, with a 2.38-fold increase shown in 24-h biofilms (*P* < 0.005). No statistical differences were observed between 12- and 24 h-biofilms (Fig. 5). Interestingly, efflux pump activity is shown to be constitutively expressed within biofilms, with no induction observed in response to azole antifungals (Fig. S4).

10.1128/mSphere.00334-18.4 FIG S4  Azole exposure does not induce Candida auris efflux pump activity. Following biofilm growth for 24 h, C. auris biofilms were treated with a range of fluconazole concentrations (0 to 256 µg/ml) for 4 h before efflux pump activity was quantified using the Ala-Nap assay. Download FIG S4, TIF file, 2.1 MB. Copyright © 2018 Kean et al. 2018 Kean et al. This content is distributed under the terms of the Creative Commons Attribution 4.0 International license.

Given the increased activity of efflux pumps in biofilms, we then assessed the contribution of these transporters to fluconazole sensitivity (Table 4). When biofilms were incubated for 12 h in the presence of fluconazole, the sessile MIC_50_ (SMIC_50_) ranged between 32 and >128 µg/ml. However, when also grown in the presence of fluconazole and an efflux pump inhibitor (EPI), the SMIC_50_ ranged between 2 and 16 µg/ml for all isolates, ranging from a 4- to 16-fold increase in susceptibility. The same trend was observed for 24-h biofilms, with the SMIC_50_ range between 64 and >128 µg/ml for fluconazole-only treatment, with 2- to 8-fold reductions observed with coincubation with the EPI (SMIC_50_, 8 to 64 µg/ml).

**TABLE 4:** Inhibition of efflux pumps increases azole susceptibility

Isolate no.	Fluconazole SMIC_50_ (μg/ml) at time:					
	12 h			24 h		
	With EPI^a^	Without EPI	Fold change	With EPI	Without EPI	Fold change
NCPF8971	16	64	4	16	>128	≥8
NCPF8973	2	32	16	8	64	8
NCPF8984	16	>128	≥8	64	>128	≥2
NCPF8990	8	32	4	16	64	4

a EPI, efflux pump inhibitor.

## DISCUSSION

**FIG 6:**
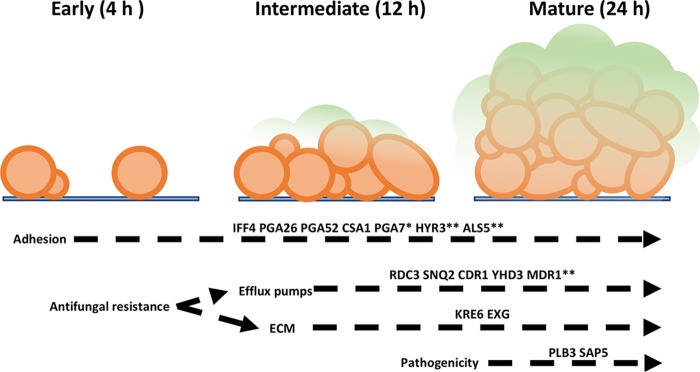
Formation and development of Candida auris biofilms. Schematic representation of the transcriptional mediators of the three main stages of C. auris biofilm development: adherence of yeast cells to surface (early phase), proliferation (intermediate phase), and maturation into a structured biofilm (mature phase).

The rapid and simultaneous emergence of the pathogenic yeast C. auris, combined with its reported recalcitrance to all three major classes of antifungals, has led to a concerted response by the medical mycology community to understand and define the mechanisms underpinning its pathogenicity and resistance. Although preliminary investigations have investigated genetic point mutations promoting resistance (7, 8), as well as a number of efflux pumps identified within its genome (17, 18), there are still substantial gaps remaining in our understanding. Moreover, irrespective of these defined chromosomally derived resistance characteristics, adaptive resistance mechanisms associated with environmental stressors are likely to be a key contributor to its success as a pathogen in both the host and the environment. We have recently reported how C. auris exhibits enhanced pathogenicity and resistance, both *in vitro* and *in vivo*, and that the biofilm phenotype is instrumental in its lifestyle (14, 16, 21, 22), and moreover, in its ability to survive and persist in the nosocomial environment, increasing the probability of causing outbreaks. We have recently reported that adherent C. auris cells display substrate-dependent susceptibility to clinically relevant concentrations of hospital disinfectants (22) and that these biofilms were shown to be resistant to chlorhexidine and hydrogen peroxide, displaying a less susceptible phenotype than C. albicans and *Candida glabrata* (21). Here, we report for the first time that efflux-based resistance mechanisms play an important role in biofilm-mediated resistance in C. auris and that conserved biofilm-related genes are temporally observed, as illustrated in Fig. 6.

To investigate this, we undertook an RNA sequencing-based approach for the analysis of C. auris biofilm development, as well as profiling genes associated with resistance and virulence mechanisms. Assembly of the transcriptome using Trinity software has allowed us to construct a specific reference for our samples of interest. Additionally, annotation via numerous methods has allowed for an in-depth functional characterization of the organism. Annotation of homologs, predicted protein domains, and gene ontological classifications further enhances our ability to interpret mechanisms that differentiate C. auris under different conditions. This annotated transcriptome has been highly instrumental in expression analysis and elucidation of virulence mechanisms of C. auris in this and forthcoming studies.

The initiation of biofilm formation depends on an initial adherence phase of colonization of a specific surface before subsequent proliferation to promote disease. A number of GPI-linked cell wall proteins were upregulated at the early biofilm time point, highlighting their role in the initial adherence stage. In C. albicans, IFF4 and CSA1 have been shown to be involved in adherence to both mucosal and abiotic substrates, as well as cell-cell cohesion (23–25). Transcriptional studies from Fox et al. identified *IFF4* as a member of a group of 10 adhesion genes that are induced at the later stages of biofilm formation and hypothesized its role in mediating cell-cell contact (26). Interestingly, an *iff4*Δ null mutant displayed decreased adhesion at an early stage of biofilm formation, as well as attenuated virulence (27). Both studies collectively highlight its function throughout biofilm formation.

In C. albicans, members of the agglutinin-like sequence (ALS) proteins play a key role in the adherence of the organism, predominantly through *ALS3* (28, 29). A recent study identified that members of this cell wall protein family detected in C. albicans are not found in C. auris (18). Our analysis revealed that orthologs of only two members, *ALS1* and *ALS5*, were represented within the C. auris transcriptome, with the latter upregulated within mature biofilms. Further examination of cell wall protein families by Muñoz et al. failed to reveal any highly expanded families (18). It is therefore likely that a less reliant ALS-dependent adherence mechanism exists for C. auris. Moreover, the gene encoding candidapepsin-5, commonly known as *SAP5* in C. albicans, was shown to be upregulated in mature biofilms. This protease is predominantly associated with its role in invasive infection (30). Indeed, studies have identified its increased expression in biofilm-associated infections (31), with *sap5*Δ/Δ strains demonstrating a less adherent phenotype, therefore highlighting its potential as a promising biofilm biomarker (32).

One of the most defining characteristics of biofilms is their recalcitrance to antimicrobial agents. As described in other *Candida* species, biofilm-associated drug resistance comprises a number of different mechanisms that coordinate with one another through the various phases of biofilm development (33). An underlying mechanism across *Candida* spp. is the upregulation of efflux pumps within biofilm-associated cells (34–36). Planktonically, C. auris isolates displayed up to 15-fold-higher ABC transporter activity than C. glabrata isolates (15), highlighting a potential intrinsic azole resistance mechanism. Ramage et al. demonstrated that expression of *CDR1* and *MDR1* was increased within mature C. albicans biofilms compared to their planktonically grown equivalents, and yet deletion of these genes had no effect on the susceptibility of mature biofilms (37). Indeed, temporal efflux pump analysis revealed that efflux pump mutants were more susceptible to fluconazole treatment than their parental strain at early phases of biofilm development (36), as also shown in other fungal pathogens, such as Aspergillus fumigatus (38). Our own temporal analysis of C. auris biofilms revealed that efflux pumps were upregulated at intermediate and mature phases of development, unlike other species, though they did not appear to be inducible following azole exposure. This is in contrast to analysis of C. glabrata biofilms exposed to azole treatment, where upregulation of genes encoding ABC transporters was observed (35). Muñoz et al. recently analyzed the transcriptional profile of planktonic C. auris in response to azole and polyene antifungals (18). After exposure of a resistant C. auris strain to amphotericin B, almost 40 genes were shown to be differentially expressed. These included genes involved in iron transport that have previously been described in C. albicans to be involved in its response to amphotericin B (39). Three of these genes (*SIT1*, *PGA7*, and *RBT5*) were shown to overlap within our own biofilm data set, indicating that these may play an additional role in our observed polyene resistance.

A further key mechanism of *Candida* biofilm resistance is the formation of the ECM, which functions to provide stability and sequestration of drugs from the biofilm, as well as protection from environmental stressors (40). Recent studies have now identified that various *Candida* spp. conserve a constitutive polysaccharide backbone that functions to impede antifungal delivery, and yet the composition of the ECM varies between species (41, 42). Although its composition remains unknown, it could be hypothesized that C. auris ECM would be similar to that of C. glabrata, given the yeast cell biofilm phenotype. Temporal analysis has shown that the formation of the ECM is time dependent and associated with intermediate and maturation phases of biofilm formation (43). Our data suggest that this is similar in C. auris, with increased expression of *KRE6* and *EXG*, a glucan-1,3-beta-glucosidase and a close ortholog of *XOG1* in C. albicans, respectively, two genes involved in matrix formation (44, 45).

Given the alarming global emergence of antifungal resistance, the requirement for new antifungals is pivotal (46). Drug efficacy and development have plateaued in recent years, yet an encouraging number of molecules remain within the antifungal pipeline (47, 48). Several studies have assessed the positive efficacy of novel compounds, including APX001, CD101, SCY078, and ceragenins, against C. auris (49–52), which may widen the spectrum of active agents against emerging resistant species. These active agents are both expansions of current drug targets, such as 1,3-β-glucan synthase inhibitors (CD101 and SCY078), and novel targets, such as GPI protein inhibitors (APX001). All of these compounds demonstrated significant *in vitro* activity against planktonic forms of C. auris, with APX001 also demonstrating enhanced *in vivo* efficacy compared to anidulafungin (51, 53). Although these preliminary data are very promising, there are limited studies evaluating their effect against sessile C. auris. The 1,3-β-glucan synthase inhibitor SCY078 was shown to significantly reduce biofilm thickness and metabolic activity after a prolonged 48-h exposure (54). Furthermore, the CSA-44 and CSA-131 ceragenins, a class of antimicrobial peptides, also demonstrated antibiofilm activity, although the concentrations needed were 4- to 64-fold greater than the planktonically active equivalent (52). APX001 is a first-in-class compound that acts by blocking GPI synthesis through inhibition of the GPI-anchored cell wall transfer protein 1 (Gwt1). Although no such studies have been performed, perhaps then APX001 is the most attractive antibiofilm target, given our identified function of GPI-anchored proteins in C. auris biofilm formation.

Given that we can now genetically manipulate this pathogenic yeast (55, 56), future work analyzing the functional roles and processes of specific genes and proteins will further enhance our understanding of biofilm-associated pathogenicity and resistance. Unraveling the key factors that regulate the transcriptional network that exists for C. auris, similar to those studies in C. albicans and Candida parapsilosis (26, 57), may provide translational insights into novel avenues for therapeutic targets for biofilm-associated infections. We have shown that efflux pumps are important during biofilm development, and this may explain why this seemingly innocuous yeast is able to survive, persist, and cause continued problems within the hospital setting.

## MATERIALS AND METHODS

### Microbial growth and standardization.

Four C. auris clinical isolates were used throughout this study (NCPF8971, NCPF8973, NCPF8984, and NCPF8990) (58). Isolates were stored in Microbank vials at −80°C prior to use, before they were subcultured onto Sabouraud dextrose agar (SAB [Sigma, Dorset, United Kingdom]) and incubated at 30°C for 48 h. Isolates were propagated overnight in yeast peptone dextrose (YPD) medium (Sigma, Dorset, United Kingdom), before washing with centrifugation as previously described (59). Cells were then standardized to 1 × 10^6^ cells/ml in RPMI 1640 medium, and biofilms were grown in microtiter plates, 75-cm^2^ tissue culture flasks, or Thermanox coverslips for 4, 12, and 24 h at 37°C.

### Characterization of biofilm formation.

Isolates were standardized as described above and grown for 4, 12, and 24 h at 37°C. Following growth, biofilms were washed with phosphate-buffered saline (PBS; Sigma, Dorset, United Kingdom), and biomass was quantified using the crystal violet assay, as previously described (59). In addition, biofilm composition was analyzed using propidium monoazide (PMA) quantitative PCR (qPCR), a method able to differentiate live cells from a population (60). Samples were prepared as previously described (60), before sonication in 1 ml of PBS at 35 kHz for 10 min in an ultrasonic water bath to remove and disaggregate the biofilm (61). After sonication, samples were incubated in the dark with 50 µM PMA (Cambridge BioScience, Cambridge, United Kingdom) for 10 min to allow uptake of the dye. All samples were then exposed for 5 min to a 650-W halogen light before DNA was extracted using the QIAamp DNA minikit, per the manufacturer’s protocol (Qiagen, Crawley, United Kingdom). One microliter of extracted DNA was then added to a master mix containing Fast SYBR Green master mix, RNase-free water, and 10 µM C. auris-specific forward and reverse primers (forward, CGCACATTGCGCCTTGGGGTA; reverse, GTAGTCCTACCTGATTTGAGGCGAC) (62). Real-time qPCR was then used to enumerate the total of number of live cells from within the biofilm, using the following thermal profile: 50°C for 2 min and 95°C for 2 min, followed by 40 cycles of 95°C for 3 s and 60°C for 30 s. Colony-forming equivalents (CFE) were then calculated based upon a standard curve of serially extracted DNA ranging from 1 × 10^8^ to 1 × 10^4^ cells/ml.

### Biofilm visualization.

Biofilms were standardized and grown on Thermanox coverslips (Fisher Scientific, Loughborough, United Kingdom) as described above. At selected time points, biofilms were washed with PBS before processing for scanning electron microscopy (SEM). Biofilms were fixed in 2% paraformaldehyde, 2% glutaraldehyde, 0.15 M sodium cacodylate, and 0.15% (wt/vol) alcian blue, before being processed as previously described (59). Biofilms were then sputter coated in gold before being viewed under a JEOL-JSM-6400 microscope.

### Planktonic and sessile susceptibility testing.

Planktonic MICs (pMICs) were determined visually using the Clinical and Laboratory Standards Institute M27-A3 broth microdilution method (63). Standardized cells were treated with serial 2-fold dilutions of miconazole nitrate (0.25 to 128 mg/liter), micafungin (0.25 to 128 mg/liter), and amphotericin B (0.063 to 32 mg/liter). In addition, biofilms were grown for 4, 12, and 24 h as described above before treatment with the same concentrations as planktonic cells. Sessile MICs (sMICs) were determined using the XTT [2,3-bis(2-methoxy-4-nitro-5-sulfophenyl)-2H-tetrazolium-5-carboxanilide salt] metabolic reduction assay (64). The sMIC was calculated as the concentration leading to 80% reduction in XTT colorimetric readings in comparison to an untreated positive control.

### RNA extraction and sequencing analysis.

Following biofilm characterization, C. auris NCPF8973, originally isolated from a wound swab (14), was chosen for subsequent transcriptomic analysis. Biofilms were grown as described above in 75-cm^3^ tissue culture flasks before being washed with PBS, and biomass was dislodged using a cell scraper. The resultant biofilm biomass was then homogenized using a bead beater, and RNA was extracted using the Trizol (Life Technologies, Paisley, United Kingdom) method (65). Following extraction, RNA was DNase treated and purified using the RNeasy MinElute cleanup kit per the manufacturer’s instructions. Quality and quantity were assessed using a Bioanalyzer (Agilent, USA), where a minimum quantity of 2.5 µg and a minimum-quality RNA integrity number (RIN) value of 7.0 were obtained for each sample. Samples were then submitted to Edinburgh Genomics (http://genomics.ed.ac.uk/) before sequencing using the HiSeq 2500 Illumina sequencer. Biological triplicates were analyzed for all variables, with the exception of 4-h biofilms, for which two replicates were used due to sequencing failure.

### Transcriptome annotation and differential expression analysis.

Raw fastq reads were quality controlled using Trim Galore v0.4.5 (https://github.com/FelixKrueger/TrimGalore) to remove Illumina adapters and trim reads with a quality score lower than 20. Reads were then aligned to the RefSeq genome sequence B8441 using HISAT2 (66). The aligned reads were then coordinate sorted, and SAM files were converted to BAM before all aligned reads were merged using SAMtools (38). The resulting aligned reads were assembled *de novo* using genome-guided Trinity v2.5.1 (66). The completed transcriptome was assessed by using the contig length distribution metrics (*N*_50_), percentage of annotation, and the third-party Benchmarking Universal Single-Copy Orthologs (BUSCO) v3 assessment program (http://busco.ezlab.org/). Annotation of candidate open reading frames (ORFs), identified with TransDecoder v5.0.2 (http://transdecoder.sourceforge.net/), was then performed using the Trinotate v3.1.0 package (https://trinotate.github.io/). Trinotate performs functional annotation of transcriptomes from the UniProt Swiss-Prot database via homology searches with the Basic Local Alignment Search Tool (BLAST) functions BLASTp for protein queries and BLASTx for nucleotide queries. Gene Ontology (GO) and Kyoto Encyclopedia of Genes and Genomes (KEGG) EggNOG identifiers were also inferred from the Swiss-Prot protein database. BLAST2GO annotation was additionally performed, which also relies upon BLAST but includes the annotation from European Bioinformatics Institute (EBI) InterPro databases. The extraction through to the annotation is summarized in Fig. 2. The reference transcriptome created by Trinity was used to create an index, and the trimmed reads were then counted and annotated against this index using Kallisto gene abundance quantification software. Gene abundance files for each sample replicate were then imported into R for differential analysis based upon the DESeq2 package. All additional statistics, analysis, and visualization were produced within R.

### Temporal efflux pump activity and inhibition.

The efflux pump activity of planktonic and sessile cells was assessed using the alanine β-naphthylamine (Ala-Nap) fluorescent assay as previously described (38). For planktonic assessment, four C. auris isolates were standardized to 5 × 10^7^ cells/ml in the assay buffer solution (MgSO_2_ [1 mM], K_2_HPO_4_ [50 mM], and 0.4% glucose, pH 7.0). For sessile cells, biofilms were grown in black flat-bottomed microtiter plates for 12 and 24 h. Following biofilm development, these were washed with the assay buffer solution. The reaction was then initiated with the addition of 100 µg/ml Ala-Nap and developed for 60 min at 37°C. Fluorescence readings were obtained every 30 s using a fluorescence plate reader at an emission/excitation wavelength of 355/460 nm. In addition, the efflux pump inhibitor (EPI; l-Phe-l-Arg-β-naphthylamine dihydrochloride) was used in combination with fluconazole to determine if antifungal activity could be enhanced. Biofilms were developed in the presence of fluconazole (128 to 0.25 mg/liter) with and without the presence of EPI at a concentration of 64 mg/liter and incubated for 12 and 24 h at 37°C. Biofilms were then washed with PBS, before viability was calculated using the XTT assay as described above.

### Statistical analysis.

Graph production, data distribution, and statistical analysis were carried out using GraphPad Prism (version 8; La Jolla, CA) and R Studio (version 1.1). For efflux pump activity experiments, data were normalized before Student’s *t* test was used to compare samples. Statistical significance was achieved if *P* was <0.05.

### Data availability.

Raw data files are deposited under accession no. PRJNA477447.
