# Supplementary figures and images for: Transcriptome Assembly and Profiling of Candida auris Reveals Novel Insights into Biofilm-Mediated Resistance
Source: mSphere. 2018 Jul 11;3(4):e00334-18. doi: 10.1128/mSphere.00334-18 (PMC6041501; doi:10.1128/mSphere.00334-18)

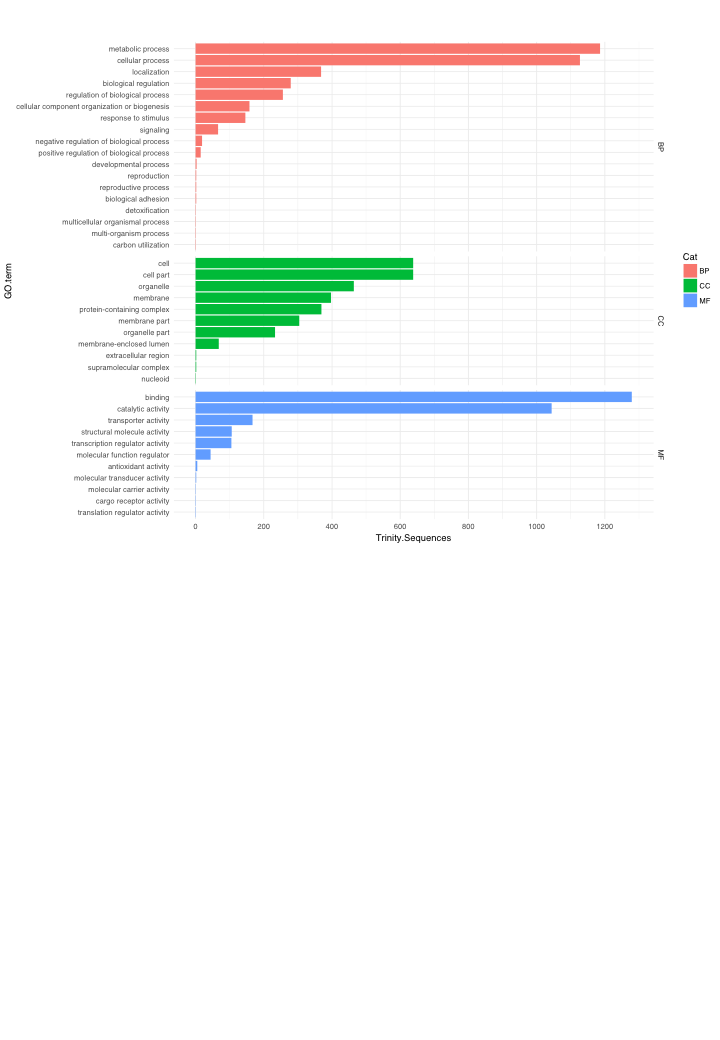

Supplement: FIG S1 [file sph004182587sf1.tif]

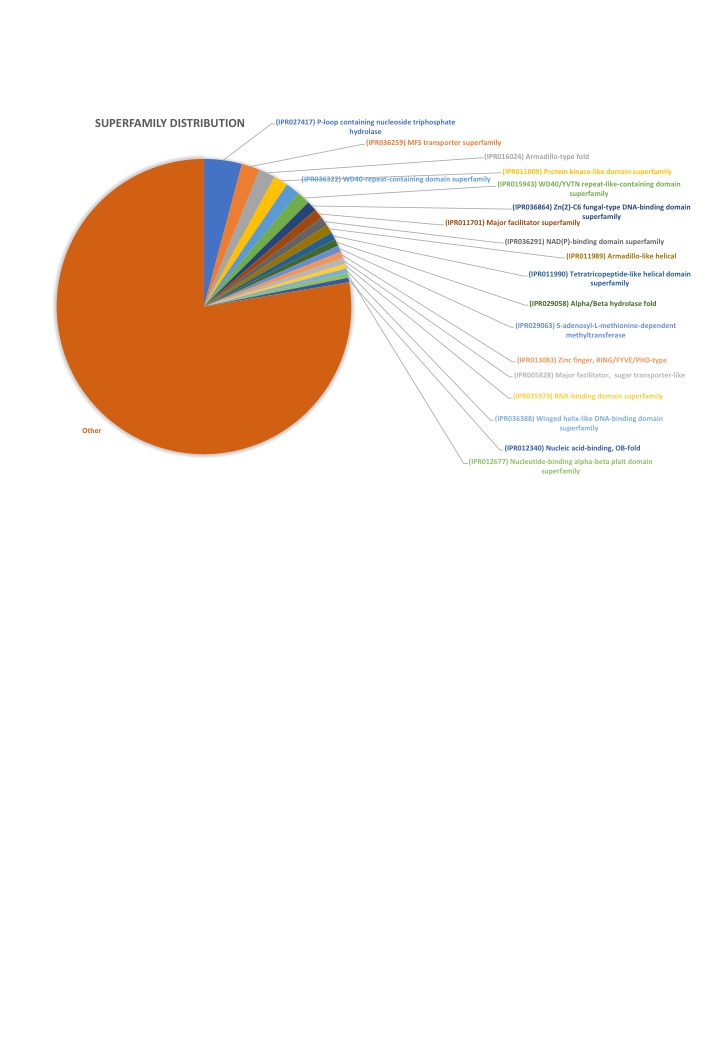

Supplement: FIG S2 [file sph004182587sf2.tif]

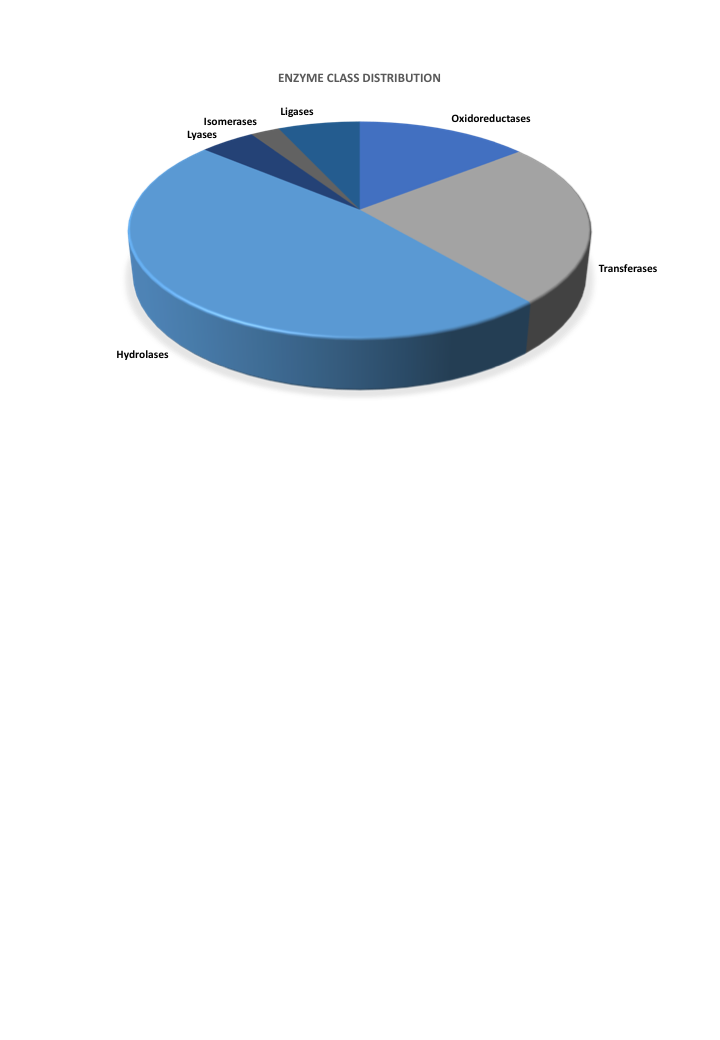

Supplement: FIG S3 [file sph004182587sf3.tif]

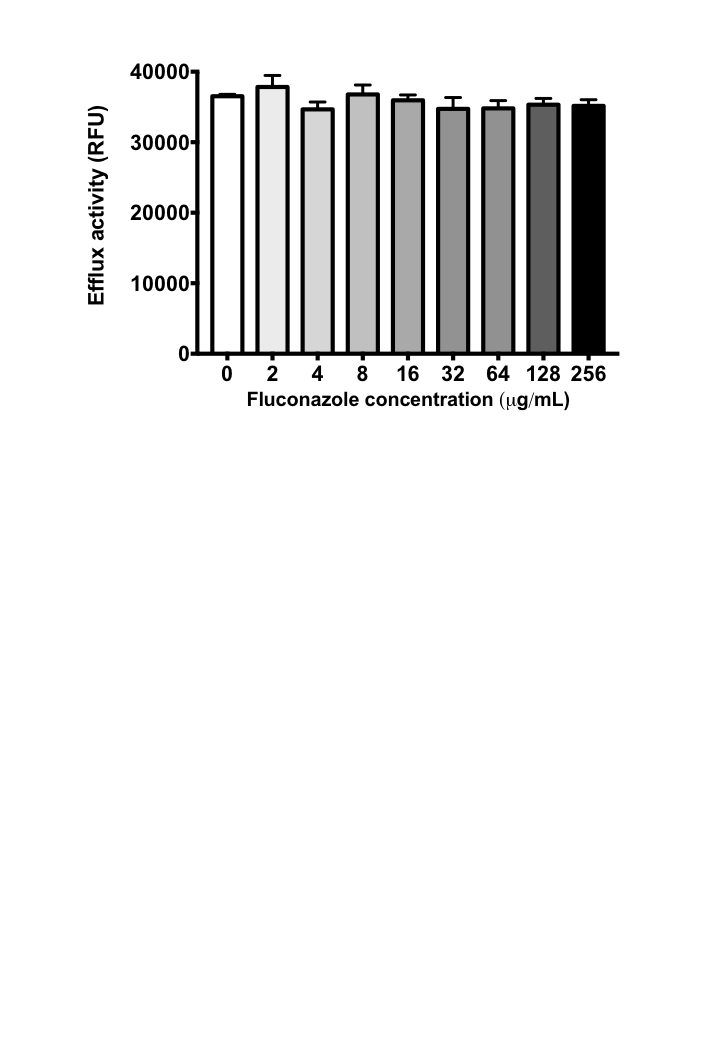

Supplement: FIG S4 [file sph004182587sf4.tif]
